# Brain-derived extracellular vesicles promote bone-fat imbalance in Alzheimer's disease

**DOI:** 10.7150/ijbs.79461

**Published:** 2023-04-29

**Authors:** Xixi Liu, Chunyuan Chen, Yaling Jiang, Meidan Wan, Bin Jiao, Xinxin Liao, Shanshan Rao, Chungu Hong, Qijie Yang, Yuan Zhu, Qianqian Liu, Zhongwei Luo, Ran Duan, Yiyi Wang, Yijuan Tan, Jia Cao, Zhengzhao Liu, Zhenxing Wang, Hui Xie, Lu Shen

**Affiliations:** 1 Department of Neurology, Xiangya Hospital, Central South University, 410008 Changsha, Hunan, China.; 2 Department of Orthopedics, Movement System Injury and Repair Research Center, Xiangya Hospital, Central South University, 410008 Changsha, Hunan, China.; 3 National Clinical Research Center for Geriatric Disorders (Xiangya Hospital), 410008 Changsha, Hunan, China.; 4 Engineering Research Center of Hunan Province in Cognitive Impairment Disorders, 410008 Changsha, Hunan, China.; 5 Hunan International Scientific and Technological Cooperation Base of Neurodegenerative and Neurogenetic Diseases, 410008 Changsha, Hunan, China.; 6 Key Laboratory of Hunan Province in Neurodegenerative Disorders, 410008 Changsha, Hunan, China.; 7 Department of Sports Medicine, Xiangya Hospital, Central South University, 410008 Changsha, Hunan, China.

**Keywords:** Alzheimer's disease, extracellular vesicles, osteoporosis, miR-483-5p, adipogenesis

## Abstract

Inadequate osteogenesis and excessive adipogenesis of bone marrow mesenchymal stem cells (BMSCs) are key factors in the pathogenesis of osteoporosis. Patients with Alzheimer's disease (AD) have a higher incidence of osteoporosis than healthy adults, but the underlying mechanism is not clear. Here, we show that brain-derived extracellular vesicles (EVs) from adult AD or wild-type mice can cross the blood-brain barrier to reach the distal bone tissue, while only AD brain-derived EVs (AD-B-EVs) significantly promote the shift of the BMSC differentiation fate from osteogenesis to adipogenesis and induce a bone-fat imbalance. MiR-483-5p is highly enriched in AD-B-EVs, brain tissues from AD mice, and plasma-derived EVs from AD patients. This miRNA mediates the anti-osteogenic, pro-adipogenic, and pro-osteoporotic effects of AD-B-EVs by inhibiting* Igf*2. This study identifies the role of B-EVs as a promoter of osteoporosis in AD by transferring miR-483-5p.

## Introduction

As global life expectancy increases, age-related disorders are becoming critical public health issues. Alzheimer's disease (AD), the most common cause of dementia, is a neurodegenerative disease characterized by progressive cognitive dysfunction 1. Deposition of insoluble amyloid-β (Aβ) and phosphorylated tau (p-tau) protein are the main pathological features of AD, affecting wide areas of the cerebral cortex and hippocampus 1, 2. Several epidemiological studies have shown that individuals with AD are more likely to develop osteoporosis 3, 4, a common degenerative disease characterized by a reduction in bone mineral density (BMD), destruction of bone microstructure, and accumulation of marrow fat 5. Magnetic resonance imaging (MRI) studies have demonstrated that the reduction in hypothalamic volume in early AD patients is strongly associated with low BMD 6. Moreover, BMD tends to decrease consistently with increasing cognitive impairment severity 7. Furthermore, people with AD have a significantly higher risk of hip fracture compared to cognitive normal controls 8, 9. Increased adipogenesis of bone marrow mesenchymal stem cells (BMSCs) at the expense of osteogenesis during aging is a key factor that leads to bone-fat imbalance and eventually results in osteoporosis 10. However, the mechanism that regulates the fate of BMSCs and induces osteoporosis under the conditions of AD pathology has not been fully elucidated.

Extracellular vesicles (EVs) are membrane-bound nanoparticles that can be released by numerous cell types 11. EVs are considered mediators of near- and long-distance intercellular communication in health and disease by transferring nucleic acids, proteins, lipids, amino acids, and metabolites to target cells 12. Compelling evidence has clarified the role of EVs in transorgan regulation. We recently found that EVs derived from the bone matrix can not only modulate the differentiation of BMSCs in bone but also regulate the calcification of vascular smooth muscle cells in vessels 13. We also showed that EVs from young osteocytes can ameliorate cognitive impairment and pathogenesis in AD mice 14. The neurons in traumatic brain injury can release osteogenic microRNA (miRNA)-enriched EVs to stimulate bone formation 15. These findings indicate the important role of EVs as exchangeable messengers of the brain-bone axis. We thus hypothesized that EVs from the AD brain may also be involved in the regulation of bone metabolism.

MiRNAs are small (20-24 nt) noncoding RNAs that can regulate cell function by inhibiting target gene expression 16. The dysregulation of miRNA expression is often found in brain diseases 17, 18 and spreads to the periphery via EVs 19, which consequently affects the biological function of the distal tissues 20. Recently, a class of miRNAs was found to be significantly upregulated in brain-derived EVs (B-EVs) from the frontal cortex and serum-derived EVs of AD patients compared to those from neurological control individuals 21. Among them, some miRNAs have been previously reported to be associated with bone metabolism, such as miR-483-5p 22, 23, miR-34c-5p 24, and miR-141-3p 25. However, it remains unclear whether these miRNAs can be delivered to bone through B-EVs and participate in the induction of bone-fat imbalance and osteoporosis under AD conditions.

In this study, we found that B-EVs from AD mice (AD-B-EVs) or from wild-type mice (WT-B-EVs) were transported to bone tissues after intracerebroventricular (ICV) or intravenous injection. AD-B-EVs and plasma EVs from AD patients (AD-P-EVs) significantly decreased osteogenesis and increased adipogenesis of BMSCs *in vitro*, and intravenously injected AD-B-EVs induced bone loss and marrow adiposity *in vivo*. WT-B-EVs did not increase BMSC adipogenesis or induce a bone-fat imbalance. Furthermore, we found that miR-483-5p was enriched in AD-B-EVs and AD-P-EVs, and also demonstrated that it mediated the anti-osteogenic, pro-adipogenic, and pro-osteoporotic effects of AD-B-EVs associated with the inhibition of *Igf2*. Our study elucidated the role of B-EVs as a mediator of neuronal control of bone metabolism during AD conditions by transferring miR-483-5p.

## Materials and Methods

### Patients

In this study, 20 cases of AD patients and 20 cases of age- and gender-matched healthy controls were recruited. All the AD patients recruited in this study underwent amyloid PET or CSF β-amyloid (A), CSF p-tau (T), and MRI scans for the brain (N) and met the ATN criteria 26. Cognitive normal healthy controls were recruited from the Health Management Center of Xiangya Hospital. Ten milliliters of peripheral venous blood were collected from all subjects for extraction of plasma EVs. Dual-energy X-ray absorptiometry (DXA; Hologic, USA) was conducted on the femoral neck of all AD patients to assess BMD. The classifications of osteoporosis, osteopenia, and normal were defined as T-Score ≤ -2.5, -2.5 < T-Score ≤ -1.0, and T-score > -1.0 respectively, according to WHO diagnostic criteria 27. Informed consent was obtained from all subjects for participation. This study was conducted in accordance with the Declaration of Helsinki and approved by the Ethical Review Board at Xiangya Hospital of Central South University in China.

### Isolation and characterization of EVs

APPswe/PS1dE9 transgenic (AD) mice were obtained from Jackson Laboratory. B-EVs were isolated from the cerebral cortex and hippocampus of 6-month-old AD mice or age- and sex-matched wild-type mice 28. In brief, the brain was dissected and placed in 12 mL cold Hibernate-A medium (A12475-01, Life Technologies). The heart and liver were dissected and placed in RPMI-1640 medium (PM150110, Procell Life Science & Technology, Wuhan). All the tissues were pre-perfused with phosphate buffered saline (PBS) to remove residual blood cells. The sample was then centrifuged at 12,000 × g for 5 min at 4 ℃. Then, the supernatant was collected and filtered through a 0.22 μm filter (Millipore, Billerica, USA) to remove cells and debris. The filtrate was transferred to Amicon Ultra-15 Centrifugal Filter Units (10 kDa; Millipore) and concentrated to 1.33 mL by centrifugation at 4000 × g and 4 °C. EVs were isolated from the ultrafiltration liquid by bottom-up Optiprep density gradient centrifugation according to a previously established protocol 29. The obtained EVs were used immediately or stored at -80 °C until use (avoiding multiple freeze-thaw cycles). The protein contents of EV samples were assessed using a BCA protein quantitative detection kit (MultiSciences, Hangzhou, China). The number and size of EVs were tested by nanoparticle tracking analysis (NTA) using a ZetaView PMX 110 (Particle Metrix, Meerbusch, Germany) analyzer according to previous protocols 14. The morphologies of EVs were detected using a Hitachi H-7650 transmission electron microscope (Hitachi, Tokyo, Japan).

### EV uptake assay

B**-**EVs were labeled with PKH26 (Cat. No MINI26; Sigma‒Aldrich) following the manufacturer's instructions. The labeled EVs (3 × 10^6^ vesicles mL^-1^) were incubated with BMSCs at 37 °C for 24 h. Then, the treated cells were washed with PBS and fixed with 4% paraformaldehyde (PFA) for 15 min. The cytoskeleton of BMSCs was stained with AF488-phalloidin (Cat. No A12379; Invitrogen). After washing with PBS, DAPI was applied to stain nuclei. A fluorescence microscope (Carl Zeiss Axio Imager 2, Germany) was used to obtain images.

### ICV injection

For ICV injection of WT-B-EVs or AD-B-EVs, mice were anesthetized and placed in a brain stereotaxic apparatus (SA-100, Yuyan, Shanghai, China). The DiR labeled WT-B-EV or AD-B-EV solution (3 μL) was injected into the lateral ventricle of 4-month-old WT mice at the following coordinates (relative to Bregma): anterior posterior: -0.5 mm, medial lateral: 1.5 mm, dorsal ventral: 3.2 mm. We examined the tissue distribution of B-EVs at 24 h for *ex vivo* fluorescent imaging after ICV injection (3 mice per group).

### Tissue distribution of B-EVs

To examine the distribution of B-EVs after intravenous or ICV injection, the EVs were labeled with the DiR Iodide dye (Yeasen, Shanghai, China) for *ex vivo* fluorescent imaging or labeled with DiI (Yeasen, Shanghai, China) for fluorescence microscope observation. For fluorescence microscopy observation, the tissues from mice treated with DiI-labeled WT/AD-B-EVs or vehicle were fixed with 4% PFA for 24 h. Bone tissues were then decalcified in EDTA (0.5 mol L^-1^) with shaking at 4 ℃ for 1 week. All samples were immersed in 30% sucrose aqueous solution for 2 days for dehydration. After being flash frozen in liquid nitrogen, the samples were embedded in OCT compound (Sakura Finetek USA, Inc., Torrance, CA, USA). Then, tissues were sectioned into 10-μm-thick slices. DAPI (0.5 μg mL^-1^; Invitrogen) was applied to stain nuclei. All operations were performed under light-proof conditions. The mean intensity of fluorescent signals per area was measured using Image-Pro Plus 6 software.

### Cell culture

Mouse primary BMSCs were isolated from the marrow of femurs and tibias of 3-week-old wild-type C57BL/6 mice as described in previous studies 10. BMSCs were incubated in α-MEM (HyClone, Logan, USA) containing 10% fetal bovine serum (FBS; Gibco, Grand Island, USA) and 1% penicillin-streptomycin (PS; Solarbio, Beijing, China).

### Osteogenic and adipogenic differentiation assays

BMSCs were plated in 48-well plates at a density of 3.0 × 10^5^ cells mL^-1^ for osteogenic induction and 6.0 × 10^5^ cells mL^-1^ for adipogenic induction. Twenty-four hours later, the culture medium was replaced with fresh osteogenic or adipogenic medium (Cyagen Biosciences, Guangzhou, China) supplemented with B-EVs (100 μg mL^-1^ at the protein level) from different groups (WT-B-EVs, AD-B-EVs, or an equal volume of vehicle). The medium was replaced with fresh medium containing the corresponding supplements every 48 h for osteogenic induction, or 72 h for adipogenic induction. After differentiation for 3 days, the total RNA of the differentiated BMSCs was extracted and the expression of pro-osteogenic/anti-adipogenic transcription genes related to osteogenesis or adipogenesis was assessed by qRT-PCR. After differentiation for 8 days, the cells were stained with ARS solution (Cyagen, Suzhou, China) to evaluate matrix mineralization. After differentiation for 12 days, the cells were stained with ORO solution (Cyagen, Suzhou, China) to detect lipid droplet formation. The percentages of ARS- and ORO-positive areas were analyzed from five random visual fields for each biological replicate and three biological replicates for each group. All *in vitro* induction experiments were repeated 3 times.

### Animals and treatment

Approval for animal care and experiments was obtained from the Ethical Review Board at Xiangya Hospital of Central South University. Four-month-old C57BL/6 male mice were used in this study. For B-EV treatment, 100 μg of B-EVs (dissolved in 100 μL PBS) or an equal volume of the vehicle was injected into the mice intravenously once a week for 8 times. No mice died after the EV treatments.

### μCT analysis

The right femurs of mice were fixed with 4% PFA overnight and stored in PBS until scanning. Then, all samples were scanned by a vivaCT80 (SCANCO Medical AG, Bruettisellen, Switzerland) with a resolution of 11.4 μm per pixel, a voltage of 55 kV, and a current of 145 μA. Images were reconstructed using NRecon software and visualized using μCTVol v2.2 software. For trabecular bone analysis, the areas between 5%-15% proximal to the distal growth plate of the femurs were selected to evaluate BMD, Tb. BV/TV, Tb. N, Tb. Th, and Tb. Sp. For cortical bone analysis, 30%-40% of regions proximal to the distal growth plate in the femoral mid-diaphysis were selected to assess Es. Pm, Ps. Pm, and Ct. Th.

### Histological and immunohistochemical staining

The left femur samples were fixed with 4% PFA, decalcified in 18% EDTA for one week, and paraffin-embedded. Next, the samples were sectioned into 5-μm-thick slices. Immunofluorescence staining for perilipin A (P1998-200UL, Sigma‒Aldrich) was conducted to detect changes in bone marrow adipocytes. Osteogenic and osteoclastic activities were evaluated by immunohistochemical staining for OCN using antibodies from Servicebio (GB11233, Wuhan, China) and TRAP staining using a kit from Sigma‒Aldrich (CS0740-1KT), respectively. Adipocytes, OCN-positive osteoblasts, and TRAP-positive osteoclasts were counted from five random visual fields of distal metaphysis for each femur section. The numbers of adipocytes per area (N. AdCs/Ar/mm^2^) and the numbers of osteoblasts (N. OBs/BS/mm) and osteoclasts (N. OCs/BS/mm) were calculated.

### ELISA

The concentrations of OCN and CTX-I in serum were measured using commercial ELISA kits from Elabscience (Wuhan, China).

### Histomorphometric analysis

To perform double calcein labeling, four mice in different groups were randomly selected intraperitoneally and injected with 0.1% calcein (10 mg kg^-1^; Sigma‒Aldrich) at 10 days and 3 days before euthanasia. The femurs were obtained and fixed overnight with 4% PFA. After dehydration, methyl methacrylate-embedded specimens were prepared and sectioned into 200-μm-thick slices, which were then polished to a final thickness of approximately 50 μm. Calcein double labeling was detected using a Zeiss fluorescence microscope (Jena, Germany). Bone dynamic histomorphometric analyses for BFR/BS (μm^3^μm^-2^) and MAR (μm day^-1^) of trabecular bone were conducted with Image-Pro Plus 6 software.

### qRT-PCR

RNA was extracted from cells using TRIzol Reagent (Invitrogen) according to the manufacturer's guidelines. RNA was extracted from plasma-EVs or B-EVs using the SeraMir Exosome RNA Purification Column Kit (System Biosciences, USA). Then, 1 μg of the total RNA was used for reverse transcription using a commercial kit (Fermentas, Burlington, Canada). The reverse transcription of miRNAs was performed using the miRNA First Strand cDNA Synthesis Kit (Tailing Reaction; Sangon Biotech, Shanghai, China). qRT-PCRs were conducted on an FTC-3000 real-time PCR system (Funglyn Biotech Inc., Toronto, Canada) using FastStart Universal SYBR Premix ExTaq (Takara Biotechnology, Japan). The relative mRNA or miRNA levels were calculated by the comparative Ct (2^-ΔΔCT^) method using GAPDH or U6 for normalization, respectively. The amplifications of the miRNAs were carried out by using miR-34c-5p-F, miR-141-3p-F, miR-483-5p-F, and U6-F as forward primer and unified reverse primer (URP) as a reverse primer to amplify miR-34c-5p, miR-141-3p, miR-483-5p, and U6, respectively. Primer sequences were as follows: mouse-*Ocn*: forward, 5′-CTGACCTCACAGATCCCAAGC-3′, and reverse, 5′-TGGTCTGATAGCTCGTCACAAG-3′, mouse-*Alpl*: forward, 5′- CCAACTCTTTTGTGCCAGAGA -3′ and reverse, 5′-GGCTACATTGGTGTTGAGCTTTT-3′, mouse-*Col1a1*: forward, 5′-GCTCCTCTTAGGGGCCACT-3′, and reverse, 5′-CCACGTCTCACCATTGGGG-3′, mouse-*Sp7*: forward, 5′-ATGGCGTCCTCTCTGCTTG-3′, and reverse, 5′-TGAAAGGTCAGCGTATGGCTT-3′, mouse-*Runx2*: forward, 5′-GACTGTGGTTACCGTCATGGC-3′, and reverse, 5′-ACTTGGTTTTTCATAACAGCGGA3′, mouse-*Cebpa*: forward, 5′-GCGGGAACGCAACAACATC-3′, and reverse, 5′-ACTTGGTTTTTCATAACAGCGGA-3′, mouse-*Pparg*: forward, 5′-TCGCTGATGCACTGCCTATG-3′, and reverse, 5′-GAGAGGTCCACAGAGCTGATT-3′, mouse-*Cfd*: forward, 5′-CATGCTCGGCCCTACATGG-3′, and reverse, 5′-CACAGAGTCGTCATCCGTCAC-3′, mouse-*Fabp4*: forward, 5′-AAGGTGAAGAGCATCATAACCCT-3′, and reverse, 5′-TCACGCCTTTCATAACACATTCC-3′, mouse-*Igf2*: forward, 5′-GTGCTGCATCGCTGCTTAC-3′, and reverse, 5′-ACGTCCCTCTCGGACTTGG-3′, mouse-*Gapdh*: forward, 5′-AGGTCGGTGTGAACGGATTTG-3′ and reverse, 5′-TGTAGACCATGTAGTTGAGGTCA-3′, U6: forward, 5′-CTCGCTTCGGCAGCACA-3′, mouse-miR-34c-5p: forward, 5′-CAGGCAGTGTAGTTAGCTGATTGC-3′, mouse-miR-141-3p: forward, 5′-CGCTAACACTGTCTGGTAAAGATGG-3′, mouse-miR-483-5p: forward, 5′-AAGACGGGAGAAGAGAAGGGAG-3′, human-miR-483-5p: forward, 5′-AAGACGGGAGGAAAGAAGGGA-3′, URP: 5′-TGGTGTCGTGGAGTCG-3′.

### Western blot analysis

Cell or EV protein extracts (20 μg) were separated by SDS‒PAGE (15.5% gel for IGF2 and 12% gel for other antibodies) and blotted on 0.2 μm PVDF membranes (Millipore). The membranes were blocked in 5% milk for 1 h and incubated overnight with specific antibodies against TSG101 (1:500; sc-7964; Santa Cruz), CD63 (1:500; sc-5275; Santa Cruz), CD9 (1:500; sc-13118; Santa Cruz), Tau (1:1000; ab80579; Abcam), 6E10 (1:1000; Cat. No. 803001; Biolegend), IGF2 (1:1000; ab9574; Abcam), and *β*-actin (1:1000; GB11001; Servicebio). Then, the membranes were incubated at room temperature with the respective secondary antibodies for 1 h (1:5000; Servicebio). After washing, the blots of the proteins were then examined using an enhanced chemiluminescence kit (Advansta). The integrated density of the bands was quantified using ImageJ software.

### AntagomiR transfection

AntagomiR-483-5p and negative control (NC) were synthesized by RiboBio Co. (Guangzhou, China). In brief, the WT-B-EVs or AD-B-EVs (100 μg) were transfected with antagomiR-483-5p (100 nmol; 50 nmol L^-1^), antagomiR-NC (100 nmol; 50 nmol L^-1^), or Cy3-labeled antagomiR-NC at 37 °C for 24 h. Then, the liquid was concentrated to 100 μL using Amicon Ultra-15 Centrifugal Filter Units (10 kDa; Millipore) before being added to the culture medium or incubated with 4-μm-diameter aldehyde/sulfate latex beads (Invitrogen, USA) for 15 min for flow cytometry assay. To detect the transfection rate, a FACSCANTO II (BD Biosciences) was used, and the percentage of fluorescence-positive particles was calculated using FlowJo 8.0 software.

### Cell counting kit-8 assay

A cell counting kit-8 (CCK-8) assay was used to determine cell viability. In total, 5 × 10^3^ cells were seeded in 96-well plates overnight. After incubation with treatments for 48 h, 10 μL CCK-8 dye was added to each well, and the cells were incubated for 1 h at 37 °C. Then, the absorbance was determined at 450 nm.

### Inhibition of Igf2

Mouse *Igf2* siRNA and NC siRNA were purchased from Sangon Biotech (Shanghai, China). The addition of the TT nucleotides in the 3′ overhangs were designed to increase siRNA efficiency in target RNA degradation 30. The siRNA sequences were as follows: si-*Igf2*: 5′-CUGAUCGUGUUACCACCCAAATT-3′; si-NC: 5′-UUCUCCGAACGUGUCACGUTT-3′. BMSCs were transfected with siRNA (50 μmol) using a ribo FECT^TM^CP Transfection Kit (RiboBio, Guangzhou, China) according to the manufacturer′s instructions. In brief, BMSCs were plated in 48-well plates and allowed to adhere overnight. The siRNA was incubated with transfection reagent for 10 min at room temperature before being added to fresh complete medium without antibiotics. Then, the cells were incubated with fresh complete medium without antibiotics containing transfection complex or vehicle for 6-8 h at 37 °C, and the medium was then replaced with fresh osteogenic or adipogenic medium.

### Statistical analysis

Statistical analysis was carried out using SPSS 26.0 (IBM Corp., Armonk, NY) and Prism 8.3 (GraphPad Software Inc., San Diego, CA). All data are presented as the mean ± SD. An unpaired and two-tailed Student′s t-test was used to analyze the differences between two groups. Statistical analysis of multiple-group comparisons was performed using one-way analysis of variance (ANOVA), followed by the Bonferroni post hoc test to assess the significance of differences between two groups. The linear regression model was used to analyze the relationship between the serum level of miR-483-5p and BMD (T-Score of the femoral neck) in AD patients. *P* values < 0.05 were considered to be statistically significant.

## Results

### B-EVs are internalized by BMSCs and transported to the bone

**Figure 1:**
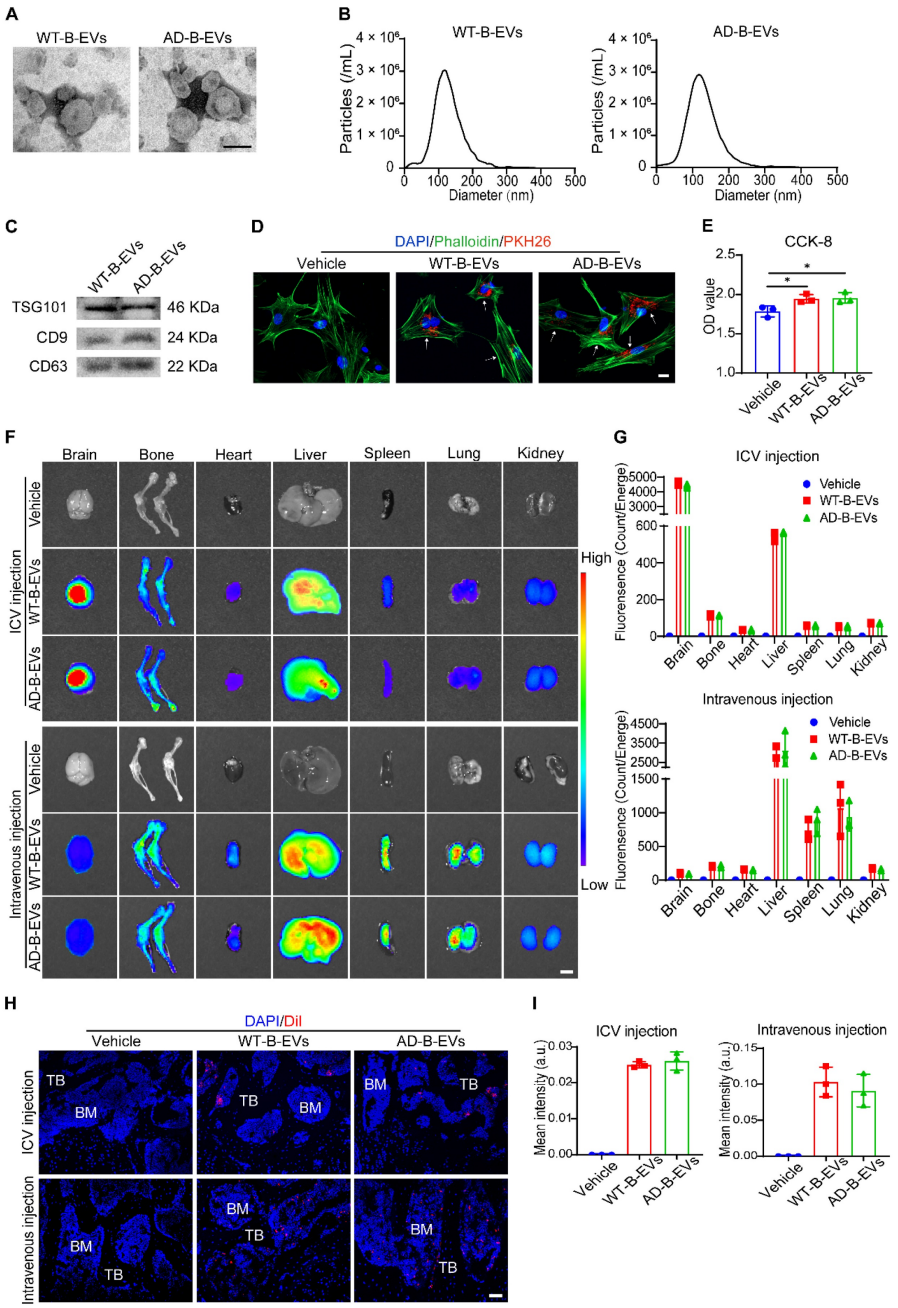
**Identification and distribution of B-EVs. (A)** Morphology of WT- and AD-B-EVs under transmission electron microscopy. Scale bar: 100 nm. **(B)** Particle size distribution of WT- and AD-B-EVs. **(C)** Western blot analysis of exosomal markers in WT- and AD-B-EVs. **(D)** BMSC phagocytosis of PKH26-labeled WT- and AD-B-EVs under fluorescence microscope. Scale bar: 20 μm. **(E)** CCK-8 assay showing the viability of BMSCs after treatment with WT-B-EVs or AD-B-EVs for 48 h. n=3 per group. **(F)** *Ex vivo* fluorescent imaging and **(G)** quantification of fluorescent signals in the brain, bone, heart, liver, spleen, lungs, and kidneys from mice intracerebroventricularly or intravenously injected with DiR-labeled WT- and AD-B-EVs for 24 h. Scale bar: 5 mm. n = 3 per group. **(H)** Fluorescence microscopy images and **(I)** quantification of fluorescence intensity of femur tissue sections from mice intracerebroventricularly or intravenously injected with DiI-labeled AD-B-EVs for 24 h. TB: trabecular bone; BM: bone marrow. Scale bar: 50 μm. n = 3 per group. The data are shown as the mean ± SD. For panel **(E)**, **(G)**, and **(I)**: one-way ANOVA with Bonferroni post hoc correction. *p < 0.05.

B-EVs were extracted from the cerebral cortex and hippocampus of 6-month-old APPswe/PS1dE9 transgenic mice (AD mice) or age- and gender-matched WT mice. Although the AD mice had developed behavioral phenotypes, the effects of aging were not yet apparent and thus could be excluded 28. WT-B-EVs and AD-B-EVs showed a typical cup- or sphere-like morphology with diameters of approximately 110 nm (Figure 1A and B). Western blotting indicated the presence of TSG101, CD9, and CD63, which confirmed their exosomal properties (Figure 1C). Fluorescence microscopy showed that PKH26-labeled WT-B-EVs and AD-B-EVs were detected in the perinuclear region of BMSCs after 24 h of incubation (Figure 1D), indicating that both WT-B-EVs and AD-B-EVs could be taken up by BMSCs. Cell counting kit-8 (CCK-8) assays showed that both WT-B-EVs and AD-B-EVs promoted the proliferation and survival of BMSCs after incubation for 48 h (Figure 1E), indicating that B-EVs did not have negative effects on the viability of BMSCs.

Next, to determine whether B-EVs could be transported to the bone, the mice were intracerebroventricularly or intravenously injected with DiR- or DiI-labeled WT-B-EVs or AD-B-EVs. After 24 h, *ex vivo* fluorescence imaging revealed that DiR-labeled WT-B-EVs and AD-B-EVs were detected in the bone and other tissues. After ICV injection, the strongest signal was visible in the liver, followed by the bone, kidney, spleen, lung, and heart (Figure 1F and G).

The intravenously injected B-EVs showed a similar tissue distribution to that of AD-B-EVs and WT-B-EVs. After intravenous injection, fluorescent signals were mainly detected in the liver, spleen, and lung, followed by the bone, kidney, heart, and brain (Figure 1F and G). No significant difference in fluorescence intensity was found between the WT-B-EV- and AD-B-EV-treated mice (Figure 1G). The distribution of DiI-labeled B-EVs was also observed in bone and other tissues under a fluorescence microscope (Figure 1H; Figure S1A), and there was no significant difference in the intensity of DiI signals between the two groups (Figure 1I and Figure S1B). Collectively, these findings suggest that both AD-B-EVs and WT-B-EVs can be transported across the blood‒brain barrier (BBB) to reach the distal bone tissue with equal targeting ability.

### AD-B-EVs and AD-P-EVs shift BMSC fate from osteogenesis toward adipogenesis

**Figure 2:**
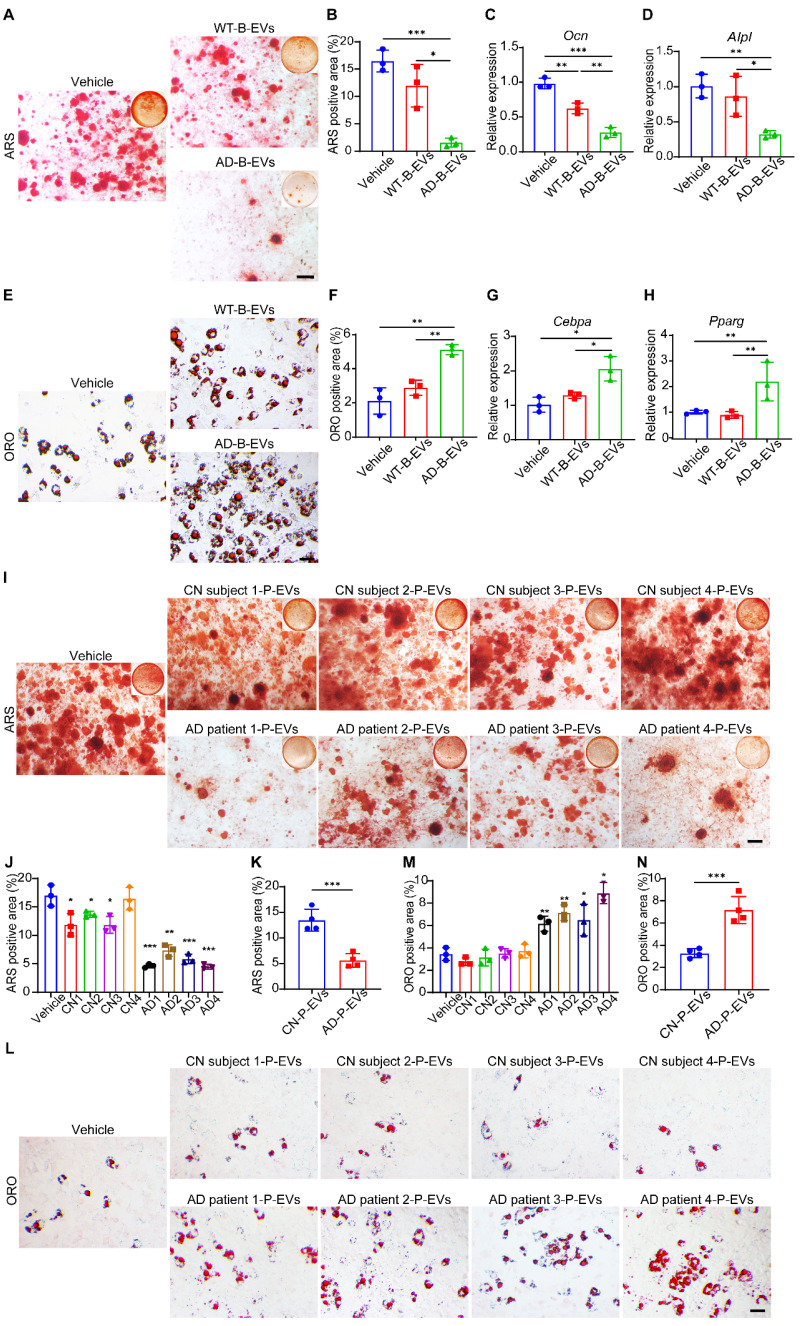
**AD-B-EVs and AD-P-EVs shift BMSC fate from osteogenesis toward adipogenesis. (A)** Alizarin Red S (ARS) staining of mineralized nodules of BMSCs receiving treatments of vehicle, WT-B-EVs, or AD-B-EVs under osteogenic inductive conditions. Scale bar: 100 μm. n = 3 per group. **(B)** The percentages of ARS-positive staining areas in **(A)**. **(C, D)** qRT-PCR analysis of the genes related to osteogenesis (*Ocn* and *Alpl*). **(E)** Oil red O (ORO) staining of BMSCs receiving treatments of vehicle, WT-B-EVs, or AD-B-EVs under adipogenic inductive conditions. Scale bar: 50 μm. n = 3 per group. **(F)** Measurement of the percentages of ORO-positive area in E. **(G, H)** qRT-PCR analysis of the genes related to adipogenesis (*Cebpa* and *Pparg*). **(I)** ARS staining of mineralized nodules of BMSCs receiving treatments of vehicle, cognitively normal subject-plasma-EVs (Four donors, named CN subject 1-CN subject 4), or AD-plasma-EVs (Four donors, named AD patient 1-AD patient 4) under osteogenic inductive conditions. Scale bar: 100 μm. n = 3 per group. **(J, K)** The percentages of ARS positively stained areas were measured (**J**: Treatment of every donor's P-EVs was compared with the Vehicle group; **K**: The comparison between CN and AD group). **(L)** ORO staining of BMSCs receiving treatments of vehicle, CN subject-P-EVs, or AD-P-EVs under adipogenic inductive conditions. Scale bar: 50 μm. n = 3 per group. **(M)** and **(N)** Measurement of the percentages of ORO-positive area (**M**: Treatment of every donor's P-EVs was compared with the Vehicle group; **N**: The comparison between CN and AD group). The data are shown as the mean ± SD. For panel **(B-D)** and **(F-H)**: one-way ANOVA with Bonferroni post hoc correction. For panel **(J)**, **(K)**, **(M)**, and **(N)**: unpaired, two-tailed Student's t-test. *p < 0.05, **p < 0.01, ***p < 0.001.

Next, we compared the effects of AD-B-EVs and WT-B-EVs on the osteogenic and adipogenic differentiation of mouse primary BMSCs *in vitro*. Alizarin Red S (ARS) staining revealed that AD-B-EVs significantly suppressed the osteogenesis of BMSCs, while WT-B-EVs had no significant effect on the osteogenic differentiation of BMSCs (Figure 2A and B). Real-time quantitative polymerase chain reaction (qRT-PCR) also showed decreased expression of osteogenesis-related genes (*Ocn* and *Alpl*) in BMSCs treated with AD-B-EVs but not with WT-B-EVs (Figure 2C and D). Oil Red O (ORO) staining showed that compared with the WT-B-EVs and vehicle groups, AD-B-EVs significantly promoted BMSC adipogenesis and upregulated the expression of adipogenesis-related genes (*Cebpa* and *Pparg*; Figure 2E-H). In addition, we also tested the expression of other osteogenesis-related genes (*Col1a1*, *Sp7*, and *Runx2*) and adipogenesis-related genes (*Cfd* and *Fabp4*) in BMSCs after being treated with AD-B-EVs. AD-B-EVs treatment decreased the osteogenesis-related genes and increased the adipogenesis-related genes in trend (Figure S2A-E). These data indicate that B-EVs from AD favor BMSC adipogenesis rather than osteogenesis. Since B-EVs can be transported from the brain to blood and peripheral tissues, we further examined the effects of plasma EVs from clinical AD patients (AD-P-EVs) and cognitively normal subjects (CN-P-EVs). Four cases of AD patients and 4 cases of age- and gender-matched cognitively normal (CN) subjects were recruited from the Neurology Clinic or the Health Management Center of Xiangya Hospital under informed consent. The collected AD-P-EVs and CN-P-EVs were used to treat the osteogenic and adipogenic differentiation of BMSCs. ARS and ORO staining showed that AD-P-EVs also exhibited notable anti-osteogenic and pro-adipogenic functions compared with CN-P-EVs (Figure 2I-N), which might be due to the contribution of AD-B-EVs.

### AD-B-EVs induce bone loss and marrow adiposity

**Figure 3:**
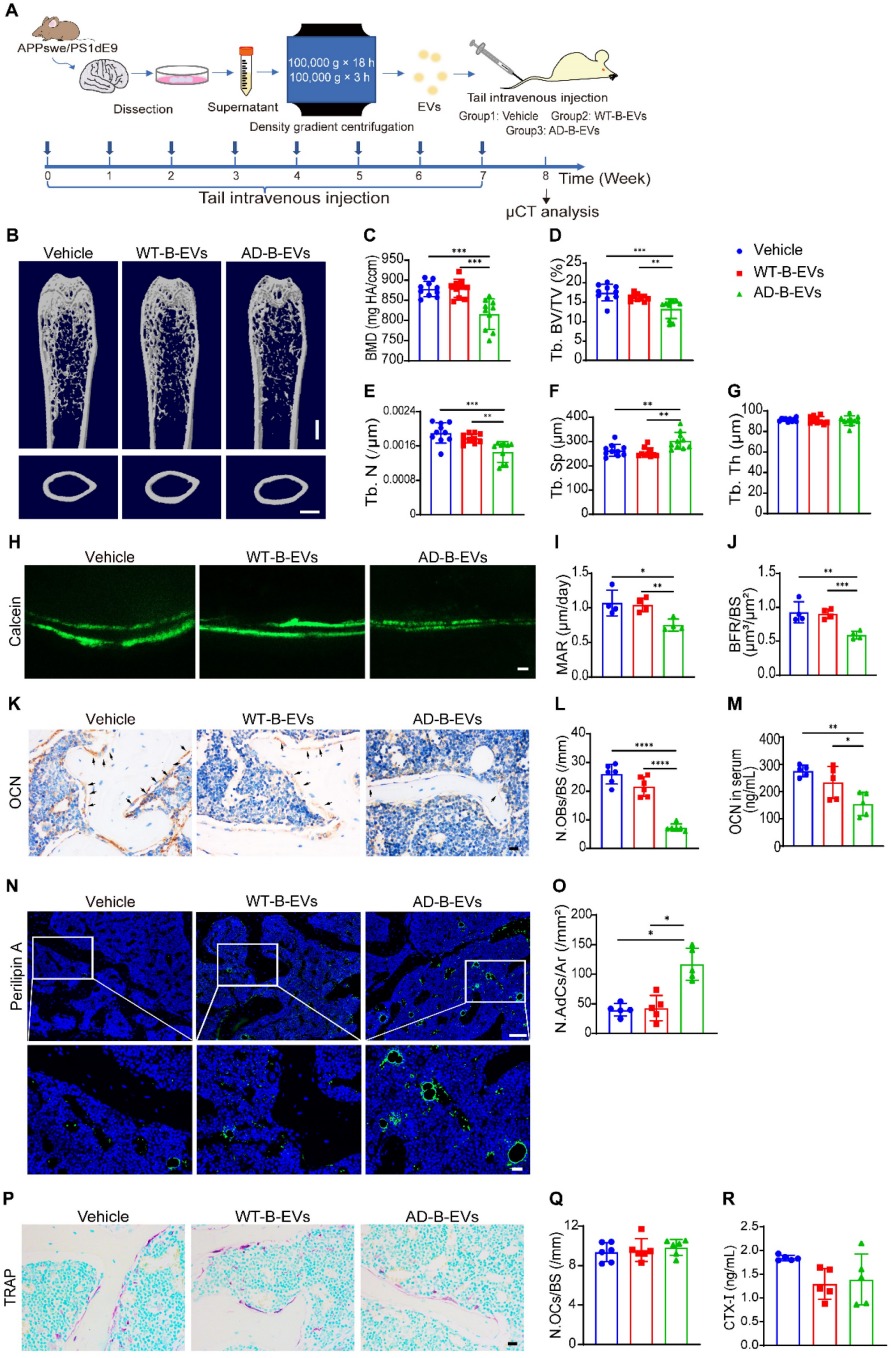
**AD-B-EVs induce bone loss and marrow adiposity. (A)** Experimental design for testing the impact of tail vein injection of AD-B-EVs on the bone. Four-month-old C57BL/6 mice were treated with vehicle, WT-B-EVs, or AD-B-EVs once a week for 8 weeks (8 times within 60 days). **(B)** Representative μCT images of femora. Scale bars: 1 mm. **(C-G)** Quantitative μCT analysis of the bone mineral density (BMD), trabecular bone volume fraction (Tb. BV/TV), trabecular number (Tb. N), trabecular separation (Tb. Sp), trabecular thickness (Tb. Th). n = 10 per group. **(H-J)** Calcein double labeling of trabecular bones **(H)** with quantification of MAR and BFR/BS **(I, J)**. Scale bar: 20 μm. n = 4 per group. **(K)** Representative OCN-stained sections and **(L)** numbers of OCN-stained osteoblasts (N. OBs) on trabecular bone surface in distal femurs. Scale bar: 20 μm. n = 6 per group. **(M)** ELISA for serum OCN. n = 5 per group. **(N)** Immunostaining images for perilipin A in distal femurs and **(O)** quantification of adipocyte number. Scale bar: 100 μm (Perilipin A), 20 μm (magnified images). n = 5 per group. **(P)** Representative TRAP staining images. **(Q)** Quantitative analysis of the numbers of TRAP-positive osteoclasts. n = 6 per group.** (R)** ELISA of the serum concentration of CTX-I. n = 5 per group. The data are shown as the mean ± SD. All dot plots: one-way ANOVA with Bonferroni post hoc correction. *p < 0.05, **p < 0.01, ***p < 0.001, ****p < 0.0001.

We next explored the effects of AD-B-EVs and WT-B-EVs on bone metabolism in normal mice. Intravenous injection was adopted for B-EV treatment since ICV injection more easily causes brain trauma and it is more difficult to achieve long-term multiple treatments than with intravenous administration. Four-month-old C57BL/6 mice were intravenously injected weekly with an equal amount of vehicle, WT-B-EVs, or AD-B-EVs for 2 months (Figure 3A). Microcomputed tomography (μCT) analysis revealed that AD-B-EVs induced a significant decrease in BMD, trabecular bone volume fraction (Tb. BV/TV), and trabecular number (Tb. N), as well as a remarkable increase in trabecular separation (Tb. Sp) compared with the vehicle-treated control group (Figure 3B-G). However, WT-B-EVs had no significant effects on these parameters (Figure 3B-G). There were no significant differences in endosteal perimeter (Es. Pm), periosteal perimeter (Ps. Pm), or cortical thickness (Ct. Th) among these three groups (Figure S3A-C). Double calcein labeling analysis showed a marked decrease in mineral apposition rate (MAR) and bone formation rate per bone surface (BFR/BS) in AD-B-EV-treated mice compared with those treated with vehicle or WT-B-EVs (Figure 3H-J). Immunohistochemical staining for osteocalcin (OCN) showed a remarkably reduced number of OCN-positive osteoblasts on the surface of trabecular bones from AD-B-EV-treated mice compared to those from vehicle- or WT-B-EV-treated mice (Figure 3K and L). Consistently, ELISA for serum OCN revealed that the level of serum OCN was markedly decreased after AD-B-EV treatment (Figure 3M). Immunofluorescence staining for perilipin A revealed a significantly increased number of perilipin A-positive adipocytes within the bone marrow in the AD-B-EVs group compared with the other two groups (Figure 3N and O). These findings indicate that AD-B-EVs can induce bone loss and marrow adiposity in mice.

We also examined the effects of AD-B-EVs and WT-B-EVs on osteoclast activity *in vivo*. Tartrate-resistant acid phosphatase (TRAP) staining revealed that there was no significant difference in the number of osteoclasts between the AD-B-EV, WT-B-EV, and vehicle treatment groups (Figure 3P and Q). ELISA for serum bone resorption marker C-terminal telopeptides of type I collagen (CTX-I) showed no significant difference in osteoclastic activity among these three groups (Figure 3R).

### MiR-483-5p is highly enriched in B-EVs from AD subjects and negatively correlated with bone mass in AD patients

**Figure 4:**
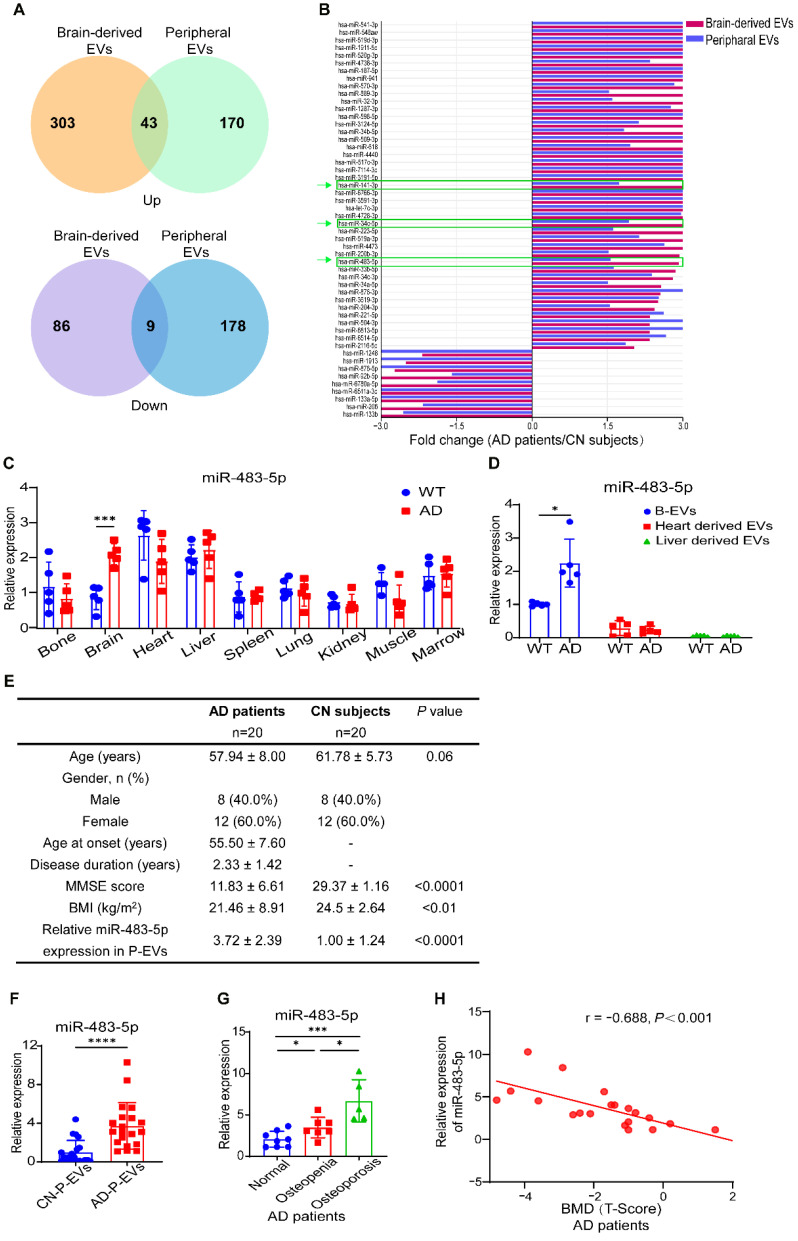
**MiR-483-5p is highly enriched in B-EVs from AD subjects and negatively correlated with bone mass in AD patients. (A)** Venn diagram of upregulated and downregulated miRNAs in AD patients compared with CN subjects and those shared by brain-derived EVs and peripheral EVs. **(B)** Histogram showing fold changes in miRNAs shared by brain-derived EVs and peripheral EVs.** (C)** qRT-PCR analysis of miR-483-5p expression in different tissues in WT or AD mice. n = 5 per group. **(D)** Relative expression of miR-483-5p in B-EVs and EVs derived from heart and liver. n = 5 per group. **(E)** Characteristics of patients with AD and CN subjetcts recruited in this study. **(F)** Relative expression of miR-483-5p in human P-EVs. n = 20 per group. **(G)** Plasma exosomal miR-483-5p levels in AD patients with different bone mass. **(H)** Relationship between plasma exosomal miR-483-5p level and BMD (T-Score) of the femoral neck in AD patients. The data are shown as the mean ± SD. For panel** (C)**, **(D)**, and **(F)**: unpaired, two-tailed Student's t-test. For panel **(G)**: one-way ANOVA with Bonferroni post hoc correction. For panel** (H)**: linear regression model. *p < 0.05, **p < 0.01, ***p < 0.001.

To explore the key miRNAs in AD-B-EVs contributing to their anti-osteogenic and pro-adipogenic effects, we dutilized the public functional genomics data on the expression of miRNAs in B-EVs and peripheral EVs of AD patients 21. Expression levels higher or lower than 2-fold in brain-derived EVs and 1.5-fold in peripheral EVs were selected as differentially expressed miRNAs. A Venn diagram showed that 43 upregulated miRNAs and 9 downregulated miRNAs were shared by brain-derived EVs and peripheral EVs in AD patients compared with CN individuals (Figure 4A). As illustrated by the differentially expressed miRNAs in Figure 4B, we selected miR-483-5p, miR-34c-5p, and miR-141-3p, which were previously reported to be associated with bone metabolism 24, 31, for further validation. qRT-PCR analysis showed that miR-483-5p, but not miR-34c-5p or miR-141-3p, was significantly increased in the brain tissues and B-EVs from AD mice compared to those from WT mice (Figure 4C and D, Figure S4A and B, and D and E). To explore the main source of peripheral EVs with high expression of miR-483-5p, we measured the miR-483-5p level in all major tissues. In WT mice, the heart and liver expressed higher levels of miR-483-5p than the other tissues; while in AD mice, the brain expressed comparable levels of miR-483-5p with the heart and liver (Figure 4C). We then measured the expression of miR-483-5p in EVs derived from the heart and liver. MiR-483-5p was enriched in B-EVs rather than heart-derived EVs or liver-derived EVs in both WT and AD mice (Figure 4D). These results indicated that B-EVs highly enriched miR-483-5p and were a major player in disorders of bone metabolism. In addition, we also examined the expression levels of two signature proteins of AD, Aβ and tau, in AD-B-EVs. Western blot analysis showed that the level of T-tau was significantly increased in AD-B-EV compared to WT-B-EV, while the level of Aβ tends to decrease (Figure S5A-C).

Next, we tested the expression of these miRNAs in P-EVs from AD patients and CN subjects. In total, 20 AD patients or CN subjects were recruited for our study. The clinical characteristics of patients with AD and CN individuals are presented in Figure 4E. The enriched level of miR-483-5p in AD-P-EVs was higher than that in CN-P-EVs (Figure 4F). According to the clinical standard of bone quality, the AD patients were divided into normal (T-Score > -1.0), osteopenia (-2.5 < T-Score ≤ -1.0), or osteoporosis (T-Score ≤ -2.5) groups 14. Along with the loss in bone quality, the enriched level of miR-483-5p in AD-P-EVs was increased (Figure 4G). Moreover, the level of miR-483-5p was negatively correlated with the BMD (T-Score) of the femoral neck in AD patients (Figure 4H). In contrast, no significant differences in miR-34c-5p or miR-141-3p were found in P-EVs from patients with AD compared with the control group (Figure S4C and F). These findings suggest that miR-483-5p may be involved in the AD-B-EV-induced regulation of BMSC fate and bone metabolism.

### MiR-483-5p mediates the anti-osteogenic and pro-adipogenic effects of AD-B-EVs on BMSCs

**Figure 5:**
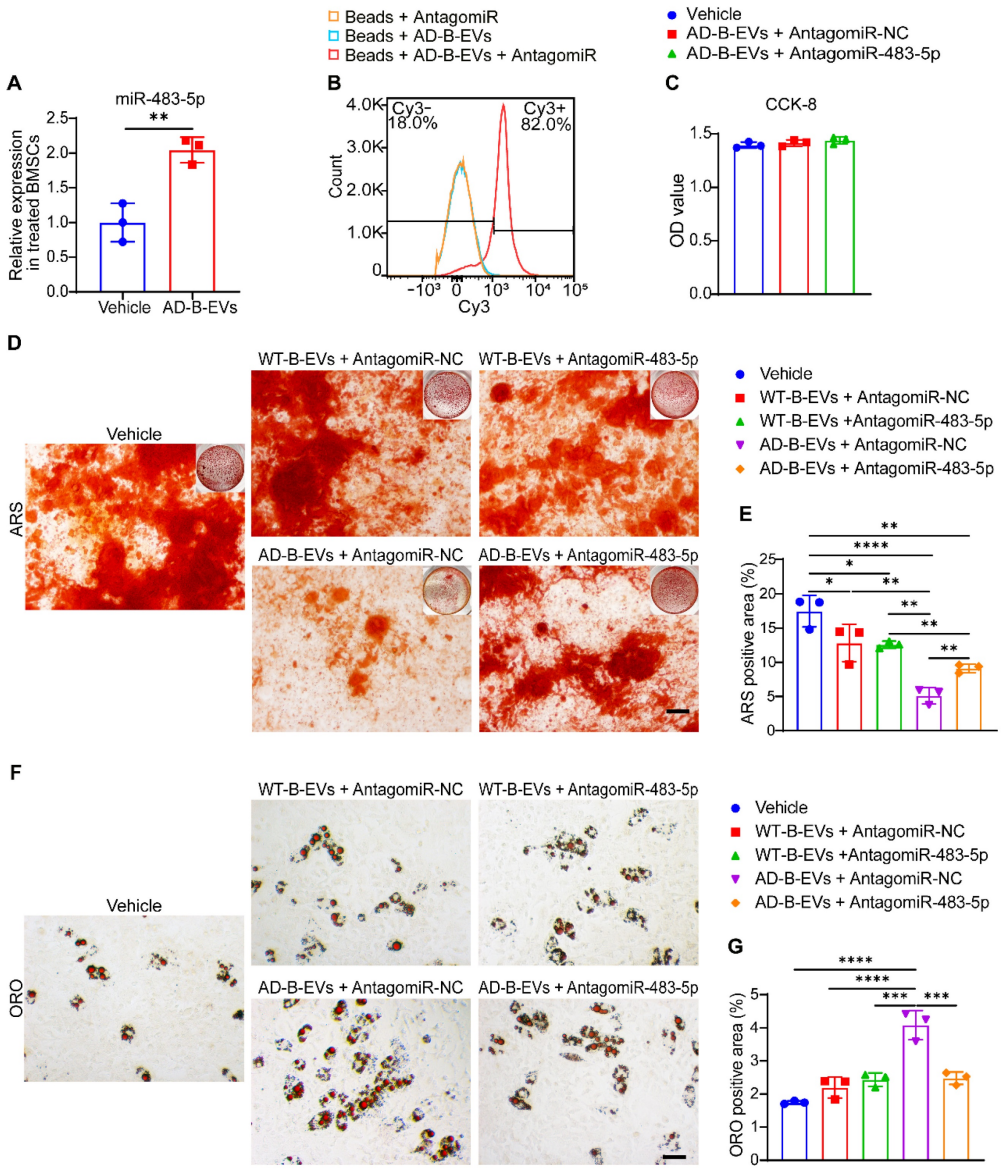
**MiR-483-5p mediates the anti-osteogenic and pro-adipogenic effects of AD-B-EVs on BMSCs. (A)** Expression of miR-483-5p in BMSCs treated with vehicle or AD-B-EVs. **(B)** Flow cytometric detection of the transfection efficiency of Cy3- labeled AntagomiR-NC to AD-B-EVs. **(C)** CCK-8 assay showing the viability of BMSCs after treated with vehicle, AD-B-EVs + AntagomiR-NC, and AD-B-EVs + AntagomiR-483-5p for 48 h. **(D)** ARS staining imaging of BMSCs treated with vehicle, WT-B-EVs + AntagomiR-NC, WT-B-EVs + AntagomiR-483-5p, AD-B-EVs + AntagomiR-NC, and AD-B-EVs + AntagomiR-483-5p under osteogenic inductive conditions. Scale bar: 100 μm. n = 3 per group. **(E)** Quantitation of the percentages of ARS positive area. n = 3 per group. **(F)** ORO staining imaging of BMSCs treated with vehicle, WT-B-EVs + AntagomiR-NC, WT-B-EVs + AntagomiR-483-5p, AD-B-EVs + AntagomiR-NC, and AD-B-EVs + AntagomiR-483-5p under adipogenic inductive conditions. Scale bar: 50 μm. n = 3 per group. **(G)** Quantitation of the percentages of ORO-positive area. n = 3 per group. The data are shown as the mean ± SD. For panel **(A)**: unpaired, two-tailed Student's t-test. For other dot plots: one-way ANOVA with Bonferroni post hoc correction. *p < 0.05, **p < 0.01, ***p < 0.001, ****p < 0.0001.

After treatment with AD-B-EVs for 3 h, the expression level of miR-483-5p in BMSCs was significantly increased compared with that in the vehicle-treated group (Figure 5A), indicating that AD-B-EVs might transfer miR-483-5p to the recipient BMSCs. To determine whether miR-483-5p contributed to the anti-osteogenic and pro-adipogenic effects on BMSCs, we used a specific antagomiR to inhibit miR-483-5p in AD-B-EVs *in vitro*. After incubation with the Cy3-labeled antagomiR-NC indicator for 24 h, flow cytometry analysis revealed that approximately 82% of AD-B-EVs were positive for Cy3 fluorescent signals (Figure 5B), suggesting that antagomiRs were able to enter AD-B-EVs. A CCK-8 assay demonstrated that the survival or growth of BMSCs was not affected by antagomiR-NC or antagomiR-483-5p pretreated AD-B-EVs (Figure 5C).

ARS staining confirmed that the inhibition of miR-483-5p by antagomiR-483-5p significantly blocked the anti-osteogenic effects of AD-B-EVs on mouse BMSCs, while pretreatment of antagomiR-483-5p did not alter the effect of WT-B-EVs on osteogenic differentiation of BMSCs compared with WT-B-EVs pretreated with antagomiR-NC (Figure 5D and E). The decrease in *Ocn* and *Alpl* expression induced by AD-B-EVs was also reversed by antagomiR-483-5p (Figure S6A and B). Moreover, ORO staining and qRT‒PCR analysis revealed that the inhibition of miR-483-5p significantly suppressed the positive effects of AD-B-EVs on lipid droplet formation (Figure 5F and G) and the expression of *Cebpa* and *Pparg* (Figure S6C and D) in BMSCs under adipogenic induction. However, inhibition of miR-483-5p in WT-B-EVs showed no significant difference in the differentiation of adipogenesis (Figure 5F and G). These findings indicate that miR-483-5p is the main mediator of the AD-B-EV-induced inhibition of osteogenesis and promotion of adipogenesis of BMSCs.

### MiR-483-5p contributes to AD-B-EV-induced promotion of bone loss and marrow adiposity

**Figure 6:**
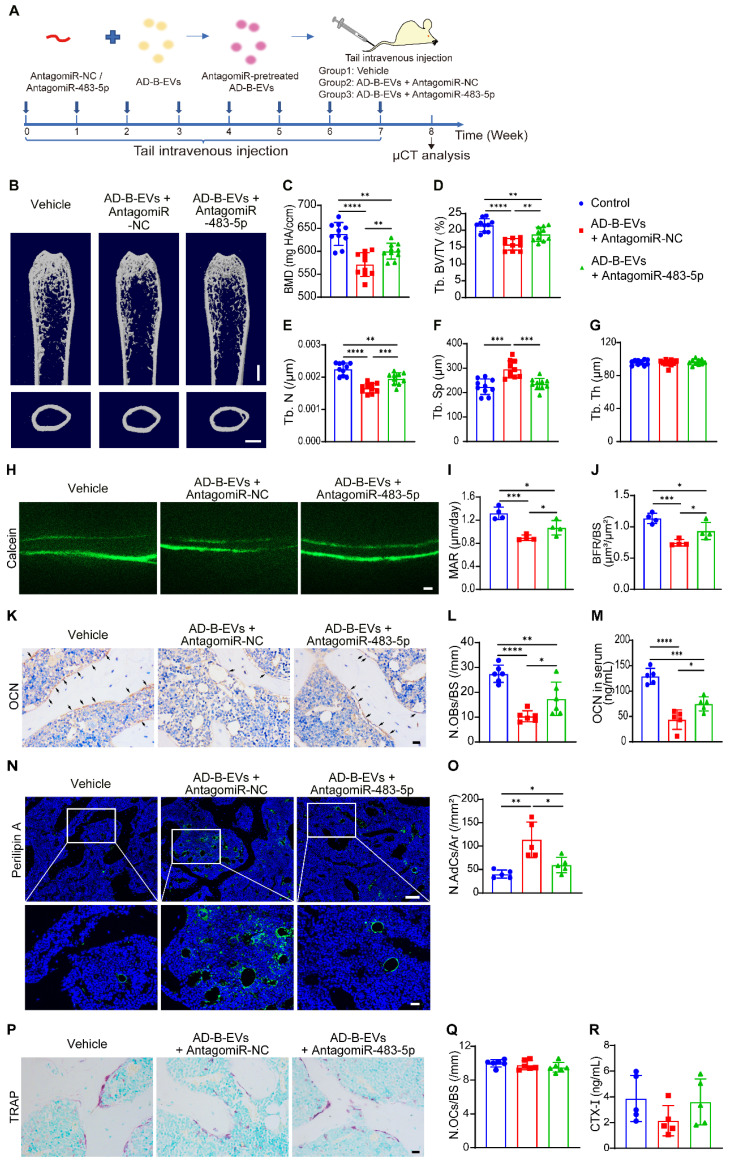
**MiR-483-5p contributes to AD-B-EV-induced promotion of bone loss and marrow adiposity. (A)** Experimental design for testing the impact of intravenous injection of antagomiR-483-5p-pretreated AD-B-EVs on bone metabolism. Four-month-old mice were treated with vehicle, AD-B-EVs + AntagomiR-NC, or AD-B-EVs + AntagomiR-483-5p once a week for 8 weeks (8 times within 60 days). **(B)** Representative μCT images of femora. Scale bars: 1 mm. **(C-G)** Quantitative μCT analysis of the BMD, Tb. BV/TV, Tb. N, Tb. Sp, and Tb. Th. n = 10 per group. **(H-J)** Calcein double labeling of trabecular bones **(H)** with quantification of MAR and BFR/BS **(I, J)**. Scale bar: 20 μm. n = 4 per group. **(K)** Representative OCN-stained sections and **(L)** numbers of OCN-stained osteoblasts on trabecular BS in distal femurs. Scale bar: 20 μm. n = 6 per group. **(M)** ELISA for serum OCN. n = 5 per group. **(N)** Immunostaining images for perilipin A in distal femurs and **(O)** quantification of adipocyte number. Scale bar: 100 μm (Perilipin A), 20 μm (magnified images). n = 5 per group.** (P)** Representative TRAP staining images. Scale bar: 20 μm. **(Q)** Quantitative analysis of the number of osteoclasts. n = 6 per group. **(R)** ELISA of the serum concentration of CTX-I. n = 5 per group. The data are shown as the mean ± SD. For all dot plots: one-way ANOVA with Bonferroni post hoc correction. *p < 0.05, **p < 0.01, ***p < 0.001, ****p < 0.0001.

We then explored whether the inhibition of miR-483-5p could abolish the effects of AD-B-EVs on bone loss and marrow adiposity. Four-month-old WT mice were intravenously injected with an equal amount of vehicle, antagomiR-NC-pretreated AD-B-EVs, or antagomiR-483-5p-pretreated AD-B-EVs once a week for 2 months (Figure 6A). As expected, μCT analysis showed that pretreatment with antagomiR-483-5p markedly reversed the AD-B-EV-induced reduction in BMD, Tb. BV/TV, and Tb. N, as well as enhancement of Tb. Sp, compared with the antagomiR-NC-pretreated AD-B-EV group (Figure 6B-F). There were no significant differences in Tb. Th, Es. Pm, Ps. Pm, and Ct. Th among these three groups (Figure 6G and Figure S7A-C). Double calcein labeling analysis showed that the AD-B-EV-induced suppression of new bone formation and mineralization was also significantly blocked by antagomiR-483-5p pretreatment (Figure 6H-J). Consistently, the decrease in OCN-positive osteoblast numbers (Figure 6K and L), the reduction in the serum levels of OCN (Figure 6M), and the increase in perilipin A-positive adipocyte numbers (Figure 6N and O) induced by AD-B-EVs were also significantly reversed, but not entirely abolished once AD-B-EVs were pretreated with antagomiR-483-5p. No significant changes were observed in the number of TRAP-positive osteoclasts or level of serum CTX-Ⅰ among the groups (Figure 6P-R). Collectively, these data further support that miR-483-5p partially contributes to the AD-B-EV-induced promotion of bone loss and marrow adiposity.

### AD brain exosomal miR-483-5p targets *Igf2* to modulate BMSC fate

**Figure 7:**
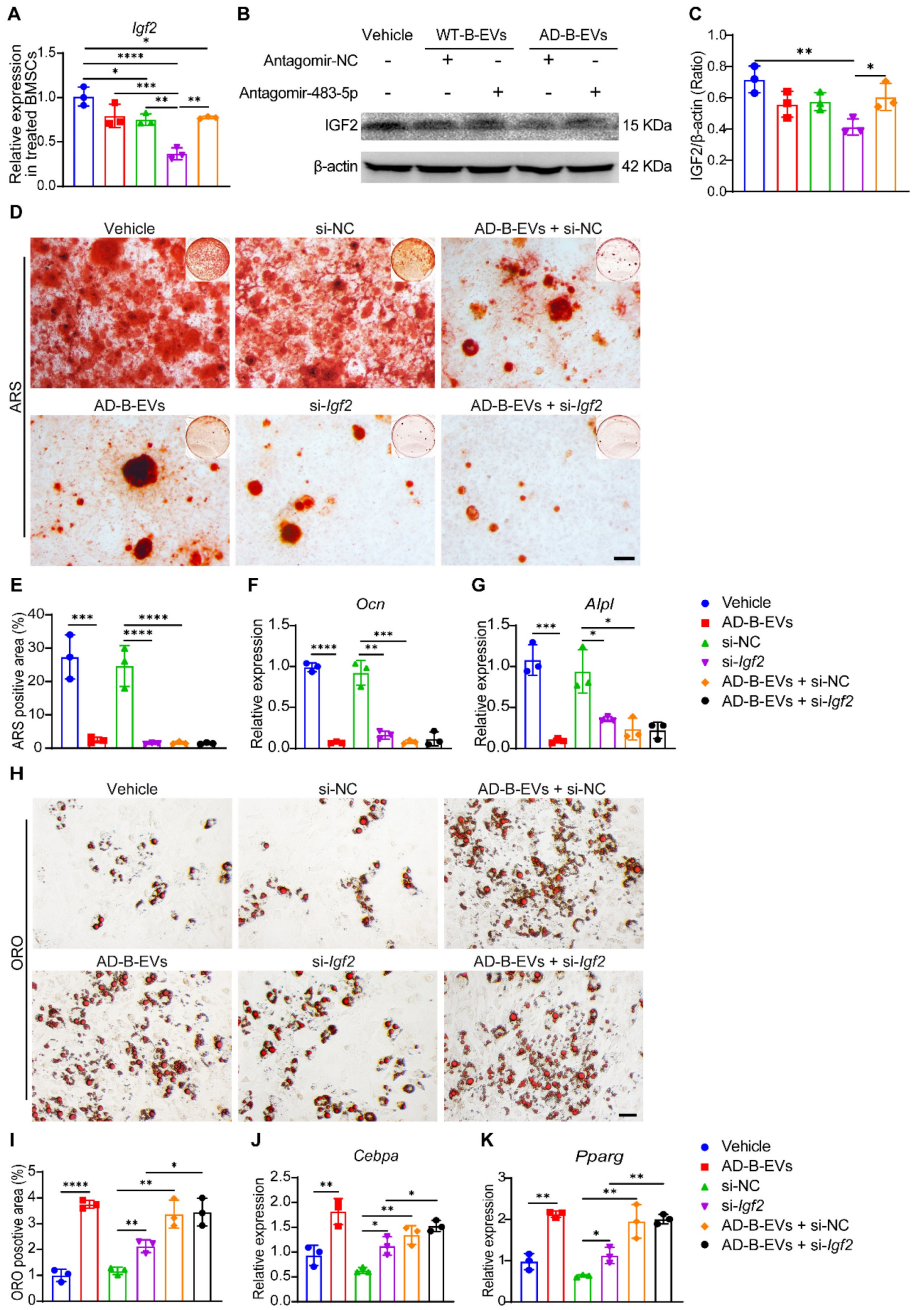
**AD brain exosomal miR-483-5p targets *Igf2* to modulate BMSC fate**. **(A)** qRT-PCR analysis of *Igf2* mRNA levels in BMSCs treated with vehicle, WT-B-EVs + AntagomiR-NC, WT-B-EVs + AntagomiR-483-5p, AD-B-EVs + AntagomiR-NC, and AD-B-EVs + AntagomiR-483-5p. **(B)** Western blot images and **(C)** relative quantification of IGF2 protein in BMSCs with different treatments. n = 3 per group. **(D)** ARS staining imaging of BMSCs receiving different treatments under osteogenic inductive conditions and **(E)** quantification of ARS-positive area. Scale bar: 100 μm. n = 3 per group. **(F, G)** qRT-PCR analysis of the genes related to osteogenesis (*Ocn* and* Alpl*). **(H)** ORO staining images of BMSCs receiving different treatments under adipogenic inductive conditions. Scale bar: 50 μm. n = 3 per group. **(I)** Quantitation of the percentages of ORO-positive area. n = 3 per group. **(J, K)** qRT-PCR analysis of the genes related to adipogenesis (*Cebpa* and *Pparg*). n = 3 per group. The data are shown as the mean ± SD. For all dot plots: one-way ANOVA with Bonferroni post hoc correction. *p < 0.05, **p < 0.01, ***p < 0.001, ****p < 0.0001.

Previous studies have demonstrated that miR-483-5p directly targets insulin-like growth factor 2 (IGF2) 32, which has been shown to regulate skeletal growth 33. qRT-PCR analysis and western blotting revealed that the gene and protein expression of *Igf2* was markedly decreased in mouse primary BMSCs treated with antagomiR-NC-pretreated AD-B-EVs, and was partially recovered by antagomiR-483-5p-pretreated AD-B-EVs (Figure 7A-C), indicating that the blockade of miR-483-5p abolishes the inhibitory effect of AD-B-EVs on *Igf2* expression. However, no significant difference in *Igf2* expression was observed in WT-B-EVs pretreated with antagomiR-483-5p compared with WT-B-EVs pretreated with antagomiR-NC (Figure 7A-C). This result suggested that miR-483-5p in WT-B-EVs was not as significantly elevated as in AD-B-EVs, thus inhibition of miR-483-5p had no significant impact on the function of WT-B-EVs. The functions of exosomal miR-483-5p were further confirmed by specific siRNA to silence *Igf2* in BMSCs. ARS staining and qRT-PCR analysis showed that treatment with si-*Igf2* or AD-B-EVs significantly suppressed BMSC osteogenesis, as demonstrated by the much lower levels of calcium nodule formation and osteogenesis-related gene expression in the si-*Igf2* group and AD-B-EVs group compared with the negative control siRNA (si-NC) group and vehicle group, respectively (Figure 7D-G). However, si-*Igf2* was unable to further suppress osteogenesis in AD-B-EV-treated BMSCs, whereas co-incubation with AD-B-EVs further induced a trend of decreases in *Ocn* and *Alpl* expression in si-*Igf2*-treated BMSCs (Figure 7D-G). ORO staining and qRT-PCR analysis revealed that *Igf2* inhibition or AD-B-EV treatment significantly enhanced lipid droplet formation and adipogenic gene expression in BMSCs under adipogenic induction, and that the combined use of si-*Igf2* + AD-B-EVs induced a higher extent of adipogenesis of BMSCs compared with si-*Igf2* but not compared with si-NC + AD-B-EVs (Figure 7H-K). These findings indicate that the anti-osteogenic and pro-adipogenic effects of AD-B-EVs on BMSCs are exerted primarily, but not entirely by inhibiting *Igf2*.

## Discussion

Osteoporosis shares many risk factors with AD, including aging 1, 27, post menopause 34, *APOE*4 genotype 35, vitamin D deficiency 36, and lack of physical activity 37 among others. However, these risk factors do not fully explain this comorbidity. Since not every patient with AD has these risk factors. For example, evidence of osteoporosis is also found in patients with mild cognitive impairment (MCI, an early stage of AD), who are much younger and do not have movement defects 38, 39. Some studies have attempted to explain this phenomenon in terms of Aβ or tau mechanisms. For example, Aβ and p-tau proteins can lead to deficiencies in Wnt/β-catenin signaling, which are also associated with osteoporotic bone loss 40, 41. In addition, animal models of AD also show an earlier phenotype of osteoporosis than WT mice 40, 42. In fact, it has long been known that top-down regulation exists between the brain and bone 43. Various neurotransmitters, such as leptin, 5-hydroxytryptamine, and neuropeptide Y, have been shown to act on the hypothalamus to regulate skeletal remodeling 10, 44, 45. Although there has been some previous research on the mechanism of AD-induced bone loss, most of this work was either *in vitro* or observational. Therefore, it is still not clear how the brain achieves the regulation of bone remodeling under AD pathology. Thus, elucidating the mechanisms responsible for AD-induced bone loss will not only aid in understanding the crosstalk between the brain and bone, but also provide potential targets for the therapy of age-related degenerative diseases.

In this study, we showed that B-EVs can be transported to the distal bone, and that there is no significant difference in bone targeting ability between AD-B-EVs and WT-B-EVs. However, only AD-B-EVs regulated the fate of BMSC differentiation from osteogenesis to adipogenesis and induced bone loss and bone marrow obesity, which was not present with WT-B-EVs. Bone remodeling homeostasis relies not only on the regulation of BMSC differentiation, but also on the regulation of osteoclast activity 46. However, in the present study, the cortical thickness and the number of TRAP-positive osteoclasts were not show significantly different between the three groups after 2 months of *in vivo* intervention with vehicle, WT-B-EVs, or AD-B-EVs. Cui et al. reported that the activity of osteoclasts was increased in Tg2576 (expressing the *APPswe* mutation) mice before 4 months of ages but decreased after 4 months of ages compared to age- and gender-matched WT mice 47. However, bone loss can still be observed in Tg2576 mice after 4 months or at even older ages. This suggests that the activity of osteoclasts may dynamically change with age in AD mice, and also indicates that the effect of osteoclast activity may not play a major role in bone loss in AD. Our findings demonstrated that AD-B-EVs induce bone loss by affecting the fate of BMSCs, rather than regulating osteoclast activity.

Next, we found that miR-483-5p was enriched in the brains of AD mice, AD-B-EVs, and AD-plasma-EVs. The level of miR-483-5p in plasma-EVs was correlated with the severity of bone loss in AD patients and mediated the anti-osteogenic and pro-adipogenic effects of AD-B-EVs, indicating that miR-483-5p may serve as a potential biomarker for osteoporosis in AD patients and might be a mediator of neuronal control of bone metabolism. Notably, miR-483-5p was also found to be overexpressed in bone samples from patients with osteoporosis 48. Our laboratory recently reported that miR-483-5p is upregulated in EVs derived from aged bone matrix, and promoted adipogenesis in BMSCs 13. This evidence further confirms our finding that miR-483-5p is an indicator of bone aging. Although miR-483-5p has been reported to be associated with bone metabolism in previous studies, it has not been investigated in the mechanism of AD-related bone loss. In the present study, we found that inhibition of miR-483-5p only partially reversed AD-B-EV induced bone loss. This may be due to the fact that AD-B-EVs can also carry other miRNAs or components that can regulate the differentiation of BMSCs. Collectively, the above results demonstrate that miR-483-5p plays an important role in the bone-fat imbalance induced by AD-B-EVs. Since Aβ and tau are key proteins in AD pathology, we examined the levels of these two proteins in AD-B-EVs. We found that the levels of T-tau were significantly increased in AD-B-EVs compared to WT-B-EVs, while the levels of Aβ tended to decrease. This result is similar to the expression of T-tau and Aβ in the cerebrospinal fluid of AD patients 26. Although previous studies have found that tau protein may promote osteoporosis by affecting the Wnt/β-catenin signaling pathway, more studies are needed to verify whether tau is one of the major molecules regulating bone metabolism in AD-B-EVs.

MiR-483-5p is located in the intronic region of the *Igf2* gene. The biological functions of miR-483-5p have been explored in many physiological and pathological processes, including angiogenesis, apoptosis, tumor progression, and cartilage differentiation 49-51. Previous studies have shown that miR-483-5p can directly target IGF2 32, 52. Interestingly, the expression of *Igf2* regulated by miR-483-5p appears to occur in an age-dependent manner 52. MiR-483-5p can act as a promoter of fetal *IGF2* or coregulate* IGF2* expression in some tumors 53. However, it does not promote adult *IGF2* expression and miR-483-5p was negatively correlated with *IGF2* expression in osteoporotic bone tissue 48. The role of *Igf2* in bone regulation has been demonstrated in animal models 54. Loss of function of Igf2 leads to thin and short bones with reduced mineralization in rodents 33. *In vitro* studies have shown that the addition of exogenous IGF2 to MSCs can induce alkaline phosphatase, osteocalcin, and bone bridging protein expression 55. In addition, IGF2 can also reduce adipogenesis in adipose precursor cells 56. Here, we demonstrated that AD brain exosomal miR-483-5p can suppress the expression of *Igf2* to modulate BMSC fate. The inhibition of *Igf2* leads to downregulation of osteogenesis and upregulation of adipogenesis in BMSCs. Interestingly, previous studies have reported that IGF2 can also reduce Aβ levels in the brain and improve behavioral deficits in AD animals 57, 58. Thus, IGF2 may not only be involved in the regulation of bone homeostasis, but also play a part in the pathogenesis of AD. This may provide a potential therapeutic target for both AD and osteoporosis.

**Figure 8:**
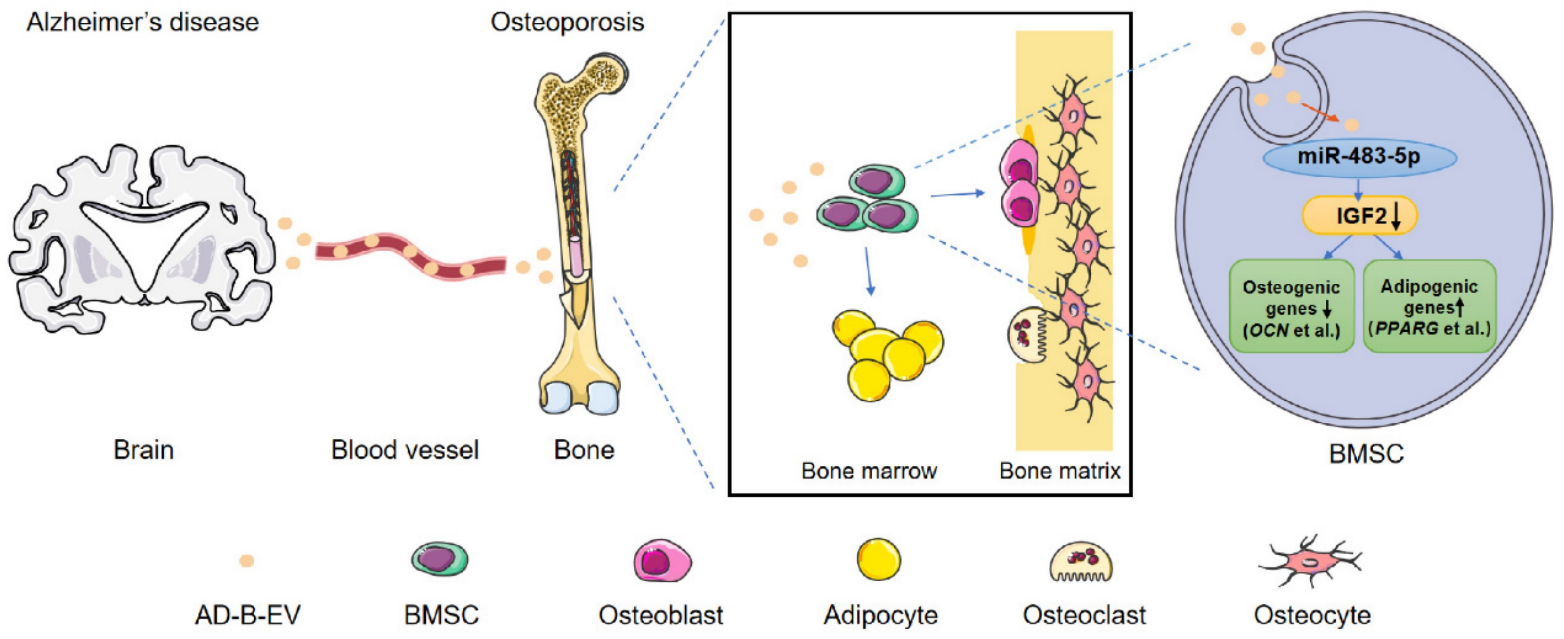
Schematic diagram shows the mode of AD-B-EVs regulating the fate of BMSC differentiation from osteogenesis to adipogenesis and inducing bone loss and bone marrow obesity. In Alzheimer's disease, B-EVs were transported to the distal bone, and target BMSCs to inhibit osteogenesis and promote adipogenesis. MiR-483-5p contributes to the AD brain-derived EVs-induced promotion of bone loss and marrow adiposity by inhibiting IGF2.

Taken together, our findings have shed light on the mechanism of AD-induced bone loss in animals and elucidated that EVs, are an important mediator of transorgan communication that can be involved in the regulation of the brain-bone axis (Fig. 8).

## Supplementary Material

Supplementary figures.
