# Supplementary material for: Brain-derived extracellular vesicles promote bone-fat imbalance in Alzheimer's disease
Source: Int J Biol Sci. 2023 Apr 29;19(8):2409–27. doi: 10.7150/ijbs.79461 (PMC10197897; doi:10.7150/ijbs.79461)
Supplement: Supplementary file 1 — Supplementary figures. [file ijbsv19p2409s1.pdf]

Supplementary material

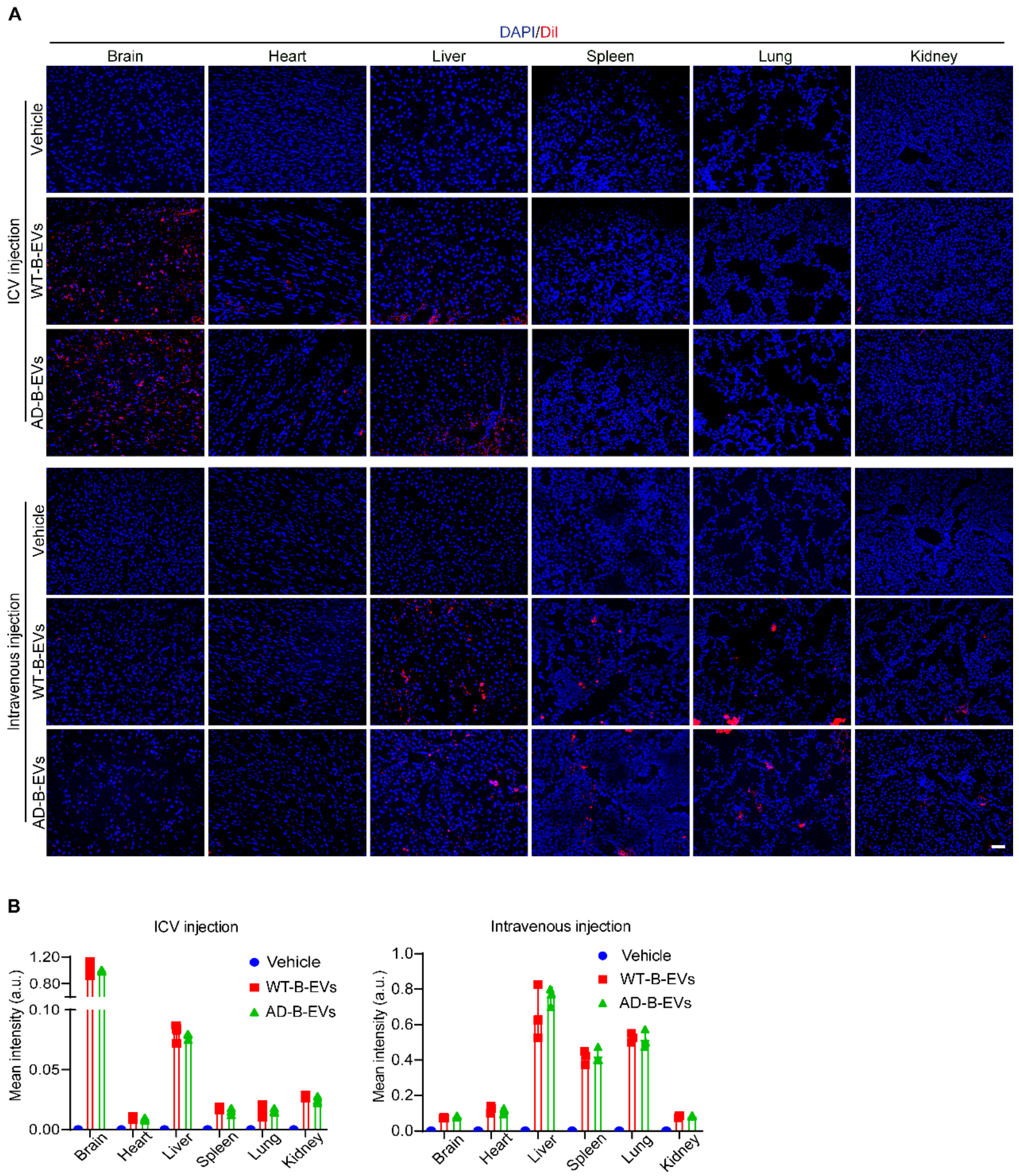

Figure S1. Distribution of DiI-labeled WT- and AD-B-EVs in the brain, heart, liver, spleen, lung, and kidney by ICV or intravenous injection. (A) Representative fluorescence images and **(B)** quantification of mean intensity of sections of brain, heart, liver, spleen, lung, and kidney sections from mice intracerebroventricularly injected with vehicle or DiI-labeled WT- and AD-B-EVs for 24 h. Scale bar: 50 μm. n = 3 per group. The data are shown as the mean ± SD. For panel **(B)**: one-way ANOVA with Bonferroni post hoc correction.

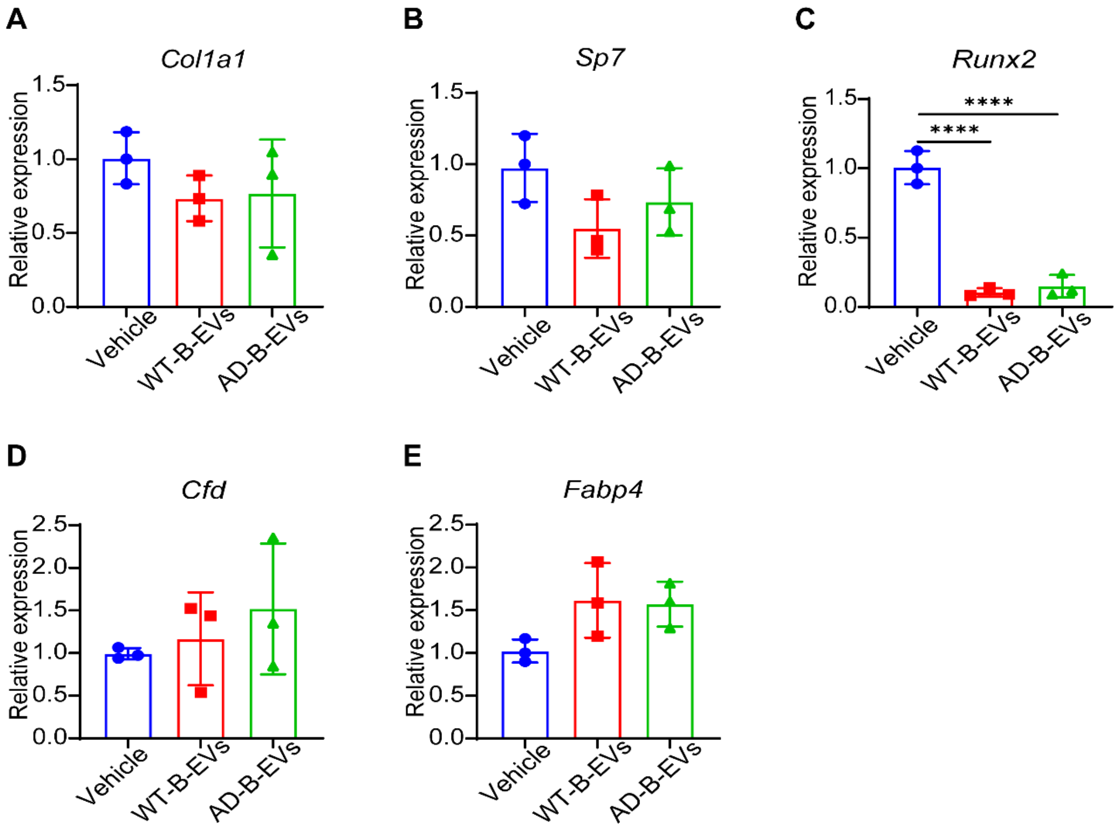

Figure S2. The expression of genes related to osteogenesis and adipogenesis in BMSCs treated with AD-B-EVs. (A-C) qRT-PCR analysis of the genes related to osteogenesis (*Col1a1*, *Sp7*, and *Runx2*). **(D, E)** qRT-PCR analysis of the genes related to adipogenesis (*Cfd* and *Fabp4*). n = 3 per group. The data are shown as the mean ± SD. For all dot plots: one-way ANOVA with Bonferroni post hoc correction. ****$p < 0.0001$.

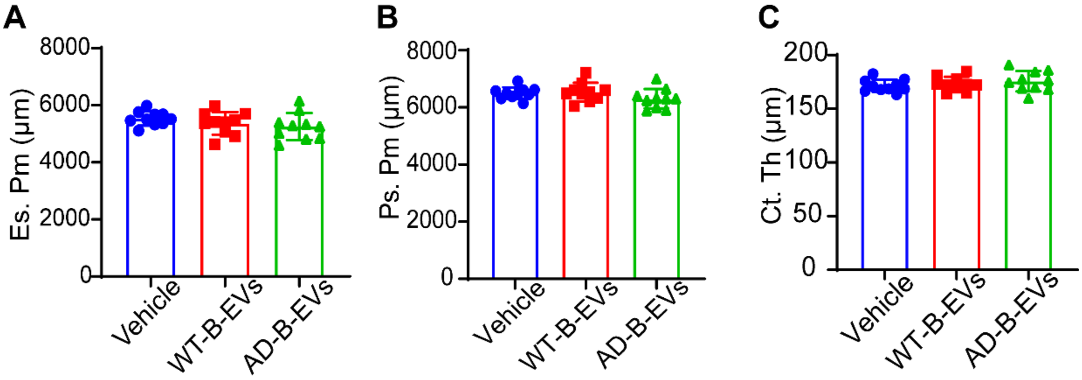

Figure S3. The effects of AD-B-EVs on osteoclast activity *in vivo*. (A-C) Quantitative μCT analysis in mice treated with vehicle, WT-B-EVs, or AD-B-EVs. Endosteal perimeter (Es. Pm), periosteal perimeter (Ps. Pm), and cortical thickness (Ct. Th). n = 10 per group. The data are shown as the mean ± SD. For all dot plots: one-way ANOVA with Bonferroni post hoc correction.

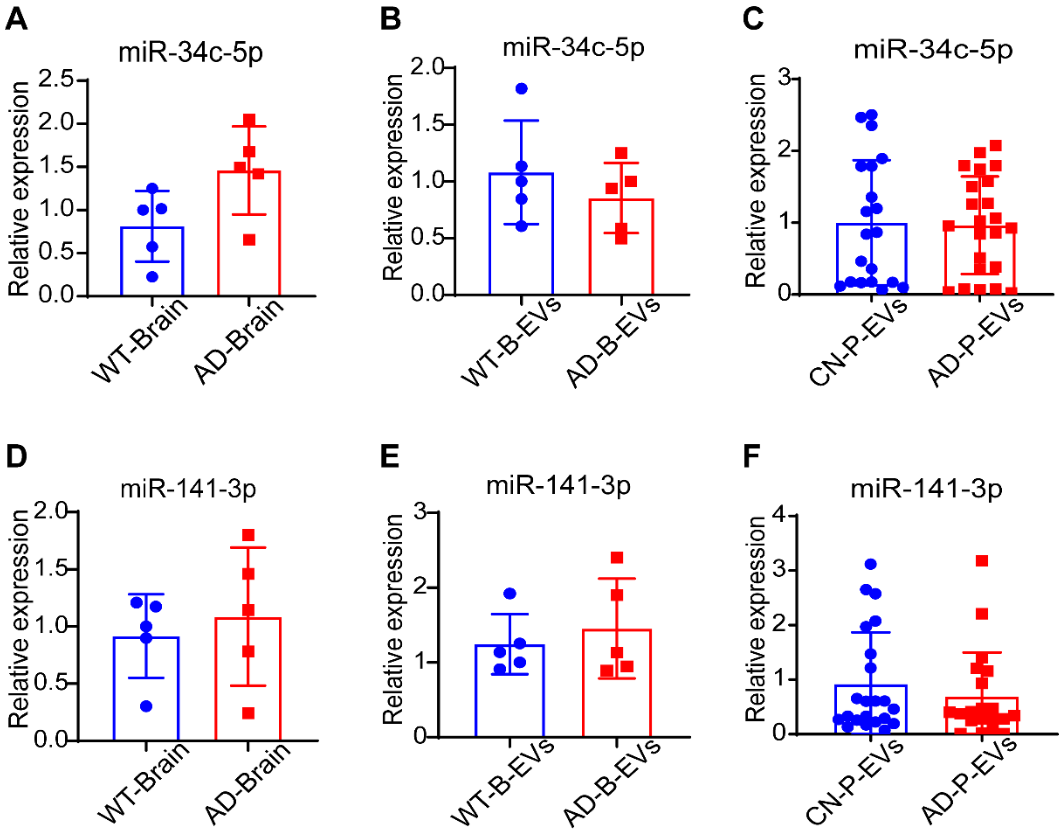

Figure S4. The expression of miR-34c-5p and miR-141-3p in mouse brain, B-EVs, and human P-EVs. (A-C) qRT–PCR analysis of miR-34c-5p expression in mouse brain, B-EVs, and human plasma EVs. n = 5 per group for mouse brain and B-EVs; n = 20 per group for human plasma EVs. **(D-F)** Relative expression of miR-141-3p in mouse brain, B-EVs, and human plasma EVs. n = 5 per group for mouse brain and B-EVs; n = 20 per group for human plasma EVs. The data are shown as the mean ± SD. For all dot plots: unpaired, two-tailed Student's *t*-test.

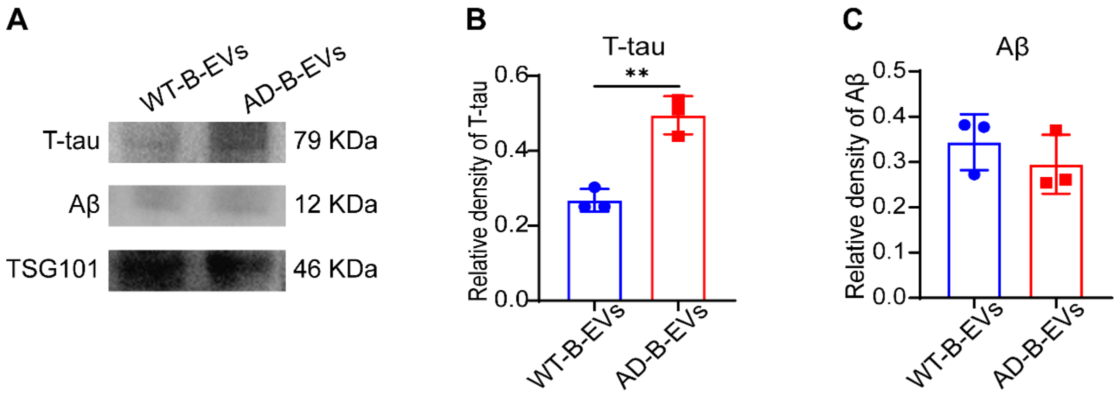

Figure S5. The expression of tau and Aβ proteins in WT-B-EVs or AD-B-EVs. (A) Western blot images and **(B, C)** relative quantification of total tau (T-tau) and Aβ proteins in WT-B-EVs or AD-B-EVs. n= 3 per group. The data are shown as the mean ± SD. For all dot plots: unpaired, two-tailed Student's *t*-test. $^{**}p < 0.01$.

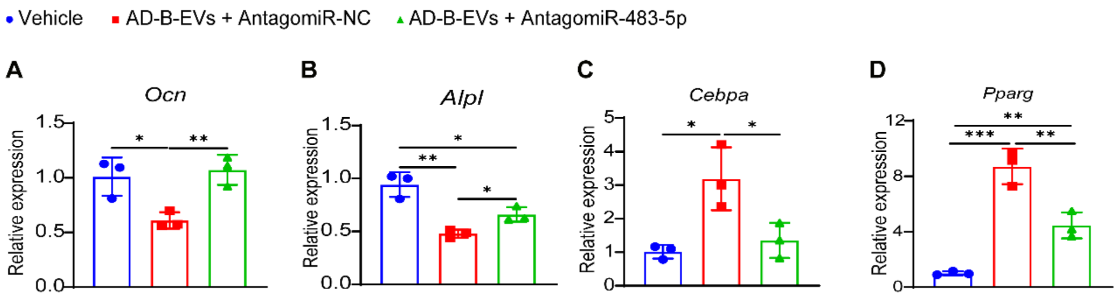

Figure S6. The expression of the genes related to osteogenesis and adipogenesis in BMSCs with different treatments. (A, B) qRT-PCR analysis of the genes related to osteogenesis (*Ocn* and *Alpl*) in BMSCs treated with vehicle, AD-B-EVs + AntagomiR-NC, and AD-B-EVs + AntagomiR-483-5p. n = 3 per group. **(C, D)** qRT-PCR analysis of the genes related to adipogenesis (*Cebpa* and *Pparg*). n = 3 per group. The data are shown as the mean ± SD. For all dot plots: one-way ANOVA with Bonferroni post hoc correction. *$p < 0.05$, **$p < 0.01$, ***$p < 0.001$.

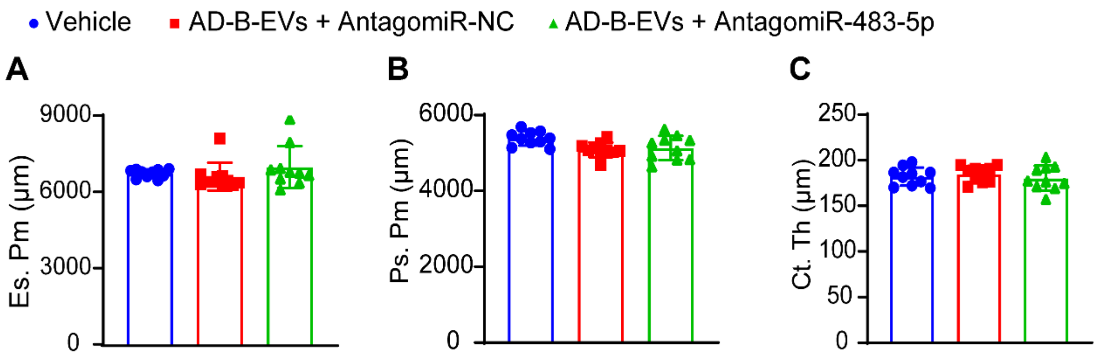

Figure S7. The effects of AD-B-EVs pretreated with antagomiR-483-5p on osteoclast activity *in vivo*. (A-C) Quantitative μCT analysis in mice treated with vehicle, AD-B-EVs + AntagomiR-NC, or AD-B-EVs + AntagomiR-483-5p: Es. Pm, Ps. Pm, and Ct. Th. n = 10 per group. The data are shown as the mean ± SD. For all dot plots: one-way ANOVA with Bonferroni post hoc correction.
